# A Review of Research Progress on Automotive Magnesium Alloy Wheels

**DOI:** 10.3390/ma19142956

**Published:** 2026-07-09

**Authors:** Meng Li, Xing Zhou, Xingmeng Zhang, Lichao An, Weijun He, Zhuang Cui, Qiu Ma, Bin Jiang

**Affiliations:** 1 CITIC Dicastal Co., Ltd., Qinhuangdao 066000, China; limeng0015@126.com (M.L.); anlichao@dicastal.com (L.A.); 2 College of Materials Science and Engineering, Chongqing University, Chongqing 400044, China; zhangxingmeng@xiaomi.com (X.Z.); cuizhuang116@163.com (Z.C.); jiangbinrong@cqu.edu.cn (B.J.); 3 Mingyue Lake Laboratory, Institute of Lightweight Materials and Engineering, Chongqing 401122, China; 4 Automobile Co., Ltd., Beijing 100176, China; maqiu1@xiaomi.com

**Keywords:** magnesium alloys, automotive wheels, lightweighting, forming processes

## Abstract

Driven by the automotive industry’s strategies for energy conservation, emission reduction, and lightweighting, magnesium alloy wheels have emerged as a key focus of research and industrialization efforts, owing to their high specific strength, excellent vibration-damping properties, and superior heat dissipation performance. This paper provides a systematic review of the performance advantages, material systems, forming processes, applications, and industrialization challenges of magnesium alloy automotive wheels. The core advantages of magnesium alloy wheels in terms of weight reduction, vibration damping, and thermal management are elaborated. The compositional characteristics, suitable processes, and performance differences between cast magnesium alloys (e.g., AZ91D, AM60B) and wrought magnesium alloys (e.g., AZ80, ZK61-Y) are outlined. The technical characteristics, microstructural and property evolution, and limitations of casting processes (gravity, high-pressure, low-pressure, and semi-solid casting), plastic forming processes (isothermal extrusion forging, backward extrusion forging, and spin forming), and hybrid processes are discussed. Combined with the case studies of magnesium alloy wheel applications in the automotive sector, this paper analyzes the core bottlenecks of magnesium alloy wheels in terms of corrosion resistance, production cost, and industrial consistency, and outlines future research directions. This paper aims to provide theoretical references and technical support for the design, manufacturing, and large-scale application of lightweight, high-performance magnesium alloy wheels.

## 1. Introduction

As global automotive emission regulations become increasingly stringent and range anxiety for new energy vehicles (NEVs) escalates, lightweighting has emerged as a core strategy for the automotive industry to improve energy efficiency and mitigate carbon emissions [1,2,3]. Driven by global carbon neutrality imperatives and circular economy initiatives, weight reduction serves not only as a means to decrease energy consumption, but also as a key enabler for sustainable automotive development. Studies demonstrate that a 10% reduction in vehicle mass can yield a 6–8% improvement in fuel efficiency [4]. For battery electric vehicles (BEVs), an equivalent weight reduction can extend the driving range by 10–14%. As the lightest metallic structural materials in current engineering applications–with a density of approximately 1.74 g/cm^3^, representing merely two-thirds that of aluminum alloys and one-quarter that of steel–magnesium alloys exhibit a unique combination of high specific strength, high specific stiffness, and exceptional vibration-damping capacities. Consequently, they demonstrate tremendous application potential across various sectors, including rail transit, electronics, and automotive manufacturing [5,6,7]. Notably, under the burgeoning trend of “giga-casting” (large-scale integrated die casting), magnesium alloys are increasingly transitioning from non-load-bearing components to critical structural members, owing to their exceptional castability and dimensional stability.

As primary unsprung rotating load-bearing components, automotive wheels offer weight-reduction benefits that significantly outperform those of sprung components (the efficiency of unsprung weight reduction is approximately 1.1 times that of sprung mass). Consequently, they play a critical role in enhancing dynamic response, reducing fuel consumption, and improving handling stability [8,9,10,11]. From the perspective of vehicle dynamics, mass reduction in wheels significantly decreases the moment of inertia, thereby shortening braking distances and improving acceleration response [12]. Compared with conventional aluminum alloy wheels, magnesium alloy wheels achieve a weight reduction of approximately 30%, while exhibiting a damping capacity roughly 15 times that of aluminum alloys and 60 times that of steel. Consequently, they significantly mitigate operational vibrations and prolong the service life of both the braking system and tires [13,14]. Furthermore, owing to their acceptable thermal conductivity, magnesium alloy wheels facilitate the rapid dissipation of friction-induced heat during braking. Coupled with the convective airflow promoted by the wheel design, this effectively mitigates the risk of thermal fade in the braking system [15,16,17,18]. These unique attributes position magnesium alloys as an optimal candidate for the lightweighting of automotive wheels. While research and application of magnesium alloy wheels have advanced considerably, several fundamental bottlenecks still impede their large-scale deployment. In terms of material systems, cast magnesium alloys (e.g., AZ91D, AM60B) have been considered promising candidates for wheel components mainly due to their ultra-low density, which enables remarkable unsprung mass reduction compared with conventional aluminum alloys. Although these Mg alloys exhibit acceptable cost-effectiveness and mold-filling capability, they possess much higher chemical activity and are more thermodynamically prone to shrinkage porosity and oxide inclusions than their aluminum counterparts. Notably, the wider freezing range of typical Mg alloy systems and the high volatility of molten magnesium make such casting defects far more harmful to structural stability and fatigue resistance under high-load service conditions than those found in aluminum wheels [19,20]. Conversely, wrought magnesium alloys (e.g., AZ80, ZK61-Y) achieve significant grain refinement via plastic deformation, thus delivering excellent mechanical properties [21]. Unlike aluminum alloys, wrought Mg alloys feature considerably higher specific strength, comparable specific stiffness, and outstanding inherent damping capacity, which effectively improve vehicle lightweighting and noise, vibration, and harshness (NVH) performance. To further overcome strength limitations and enhance the inferior high-temperature stability of conventional magnesium alloys, rare-earth (RE) elements (e.g., Gd, Y) are added to activate non-basal slip systems, tailor texture and regulate microstructural evolution, and ultimately improve the strength–toughness synergy at elevated temperatures. Nevertheless, stringent requirements for oxidation control arising from exothermic oxidation reactions, high raw material costs, and severe microstructural inhomogeneity caused by the anisotropy of hexagonal close-packed (HCP) crystal structures during processing still hinder the large-scale commercial application of RE-containing magnesium wheels, in contrast to well-established aluminum wheel materials. Fundamentally, these performance and processing bottlenecks are governed by the microstructural features and electrochemical behaviors of secondary phases. For example, the coarse, continuous *β*-Mg_17_Al_12_ eutectic networks typical of conventional AZ-series alloys act not only as brittle crack initiation sites during high-load cornering but also as aggressive local cathodes that accelerate micro-galvanic corrosion with the α-Mg matrix. Conversely, although the targeted addition of alloying elements (e.g., La, Y, Zr) can introduce thermally stable intermetallics (such as Al_11_La_3_ or the W-phase) to disrupt these continuous networks and restrict grain growth via constitutional supercooling, the resulting complex intermetallic configurations invariably impose stringent requirements for precise melt purification and multi-scale corrosion protection strategies.

Regarding forming processes, casting techniques (including gravity, high-pressure, low-pressure, and semi-solid casting) facilitate the near-net-shape forming of complex structures. Notably, advanced methods such as semi-solid casting have successfully enabled the pilot-scale production of 16-inch and 20-inch wheels, yielding substantial improvements in material utilization and component integrity. Conversely, plastic forming processes (e.g., isothermal extrusion forging, backward extrusion forging, spin forming) improve mechanical properties through dynamic recrystallization (DRX) [22]. However, the hexagonal close-packed (HCP) crystal structure of magnesium alloys dictates that room-temperature deformation is dominated by basal slip. This leads to a narrow thermomechanical processing window and the formation of strong basal textures, which inevitably induce pronounced mechanical anisotropy, complicate forming procedures, and elevate costs [23,24,25]. Furthermore, although hybrid processes (e.g., squeeze casting–isothermal forging and expansion–reduction extrusion + aging) harness the synergistic advantages of individual techniques, their complex multi-parameter coupling and regulatory mechanisms remain to be fully elucidated. Finally, under long-term service conditions, the corrosion resistance of magnesium alloys and the durability of surface coatings represent the ultimate hurdles for their large-scale industrialization.

Against this background, this review aims to provide a systematic analysis of current research findings and industrialization experiences regarding magnesium alloy automotive wheels. It first elucidates the intrinsic mechanisms governing their core performance advantages, followed by a detailed discussion on the classification and development of material systems. Particular emphasis is placed on critically evaluating the technical characteristics and microstructure-property tailoring mechanisms inherent to diverse forming processes. By dissecting the fundamental bottlenecks currently restricting industrialization and outlining future research trajectories, this comprehensive review aims to establish a robust theoretical framework and technical roadmap for the design, manufacture, and large-scale deployment of high-performance magnesium alloy wheels.

## 2. Performance Advantages of Magnesium Alloy Automotive Wheels

**Figure 1:**
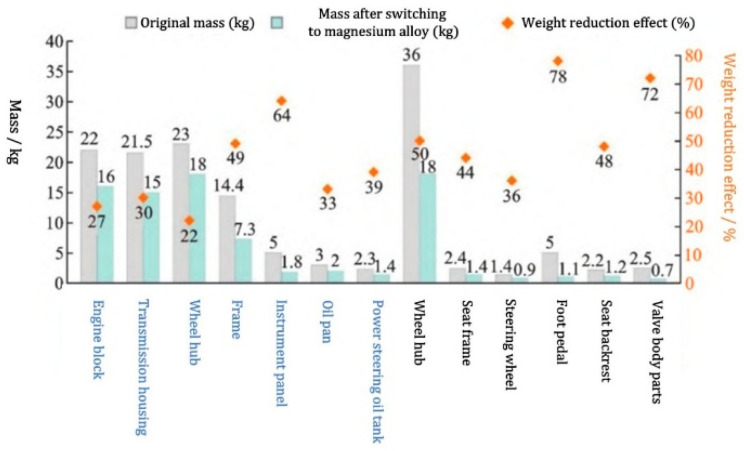
Weight reduction effect of magnesium alloys on automotive components [27].

Due to their low density, magnesium alloys are regarded as ideal materials for the lightweighting of automotive wheels [26]. Research indicates that under equivalent specifications and performance requirements, magnesium alloy wheels can achieve a mass reduction of approximately 22% compared with traditional aluminum alloy wheels (as shown in Figure 1 [27]). Jiang et al. [28] validated this advantage via finite element analysis (FEA), demonstrating that a topologically optimized magnesium alloy wheel (approx. 8.24 kg) achieves a substantial weight reduction of 26.6% compared with an aluminum alloy wheel of the same specification (approx. 11.23 kg), while rigorously meeting safety criteria for bending and radial loads. Frishfelds et al. [29] further modeled mainstream passenger vehicles, demonstrating that replacing aluminum alloy wheels with magnesium alloy counterparts reduces the single-wheel mass from 5.5 kg to 4.5 kg, representing an approximate mass reduction of 18%. While specific weight savings vary by vehicle model and design, comparative data regarding the physical properties and mass of wheels fabricated from different materials are summarized in Table 1 [30].

To satisfy stiffness and strength requirements, the cross-sectional thickness of magnesium alloy wheels may be slightly greater than that of their aluminum alloy counterparts; however, their overall mass remains significantly lower. This mass-reduction advantage directly contributes to enhanced fuel economy, extended driving range (crucial for NEVs), and improved handling responsiveness. Furthermore, the excellent specific stiffness of magnesium alloys enables the wheels to maintain their structural integrity during impact events. This effectively mitigates abnormal tire wear induced by wheel deformation, thereby potentially prolonging tire service life.

Beyond mass reduction, magnesium alloys exhibit exceptional attributes in vibration attenuation and thermal management. Specifically, their intrinsic damping performance contributes to excellent vibrational energy dissipation [31]. This exceptional damping capacity facilitates the rapid suppression of vibrations originating from the engine, drivetrain, and road surface. Consequently, it not only enhances overall NVH (noise, vibration, and harshness) performance and ride comfort, but also mitigates fatigue accumulation in onboard precision components and the wheels themselves, thereby potentially improving overall system durability [32]. Regarding thermal management, the high thermal conductivity of magnesium alloys facilitates the rapid conduction of braking-induced heat to the wheel surface for subsequent convective dissipation. This thermal efficiency is crucial for maintaining stable braking performance, preventing thermal fade and the associated loss of braking efficacy, and extending the service life of brake components.

## 3. Common Material Systems for Magnesium Alloy Wheels

**Figure 2:**
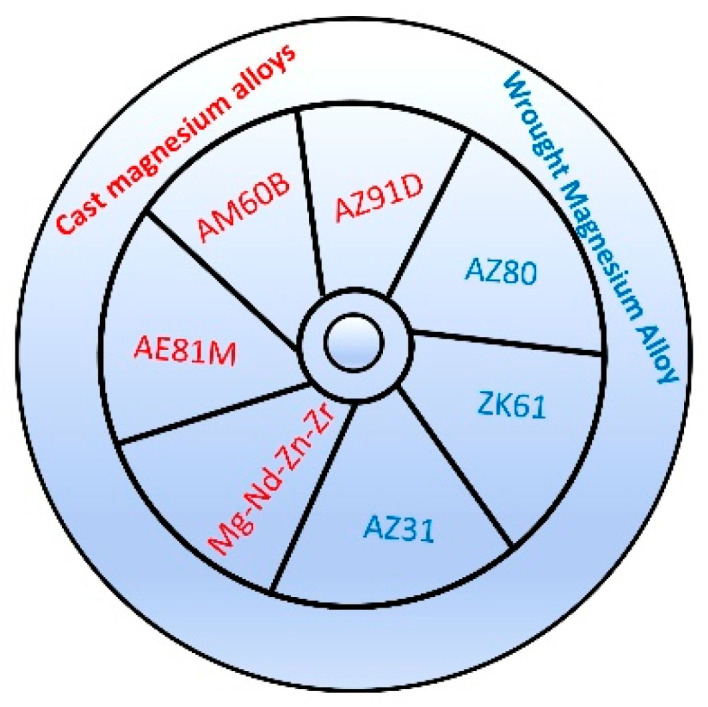
Common material systems for magnesium alloy wheels.

The selection of an appropriate material system dictates whether magnesium alloy wheels can fulfill the stringent service requirements of modern vehicles, such as load-bearing capacity, vibration damping, and corrosion resistance. At present, based on the specific forming processes and their targeted performance objectives, magnesium alloys for wheel applications are broadly classified into two primary categories: cast and wrought alloys (Figure 2).

### 3.1. Cast Magnesium Alloys

Cast magnesium alloys serve as the current mainstream choice for the near-net-shape production of wheels with complex geometries, owing to their excellent castability, high material utilization, and cost-effectiveness. Typical alloy series include the Mg-Al-Zn system (e.g., AZ91D), the Mg-Al-Mn system (e.g., AM60B), and the Mg-RE-Zn-Zr system containing rare-earth (RE) elements.

AZ91D is the most widely utilized casting grade, with its high Al content (approx. 9%) ensuring superior castability and mold-filling capacity. Research indicates that optimizing low-pressure die casting (LPDC) parameters—such as reducing the pouring velocity from 0.5 m/s to 0.3 m/s coupled with side-mold water cooling—can effectively eliminate hot-spot shrinkage cavities in the rim, thereby reducing the maximum service stress of the component by 14.4% [33]. Furthermore, multi-objective optimization of the pouring temperature (689 °C) and filling pressure (6.5 kPa) via the ant colony algorithm has been shown to refine the secondary dendrite arm spacing (SDAS) from 88.5 μm to 81.2 μm, thereby significantly enhancing microstructural uniformity [34]. Given that the intrinsic elastic modulus of magnesium alloys (approx. 45 GPa) is substantially lower than that of conventional 6061 aluminum alloy (approx. 69 GPa), the maximum static deformation of a magnesium wheel under identical loading (approx. 0.845 mm) significantly exceeds that of its aluminum counterpart (approx. 0.552 mm). To mitigate this inherent stiffness deficit, localized reinforcement of the hub’s center bore area was implemented through topology and shape optimization. Furthermore, transitioning from a standard 5-spoke double-pillar design to a multi-spoke layout effectively compensates for the material’s structural compliance. Such optimized AZ91D wheel hubs have demonstrated a reduction in maximum static stress from 41 MPa to 27 MPa, along with a marked decrease in alternating stress under radial fatigue conditions, successfully balancing mass-reduction targets with structural reliability [35].

In contrast, for applications requiring superior toughness, the AM60B alloy—characterized by a lower aluminum content—is the preferred choice. Its brittle *β*-phase (Mg_17_Al_12_) exhibits a discontinuous network distribution, offering distinct advantages in impact resistance. In material substitution design studies, a parametric optimization strategy was applied to typical lightweight wheel components; by increasing spoke width and synergistically optimizing spoke curvature (e.g., refining the critical fillet radius to *R*1 = 24.45 mm), the maximum stress was significantly reduced from 91 MPa to 66 MPa while maintaining bending fatigue strength, achieving a substantial mass reduction of approximately 29.9% [36]. To ensure forming quality for thin-walled components, vacuum die casting—integrated with the precise control of mold temperature (250 °C) and rapid injection speed (3 m/s)—further enhances near-net-shape quality and the mechanical uniformity of the wheel hub [37]. Within the Mg-Al-Mn series of high-toughness alloys, AM50A has been proven to achieve an optimal strength-toughness synergy through process optimization. For wheel components featuring complex geometries, comparative studies between conventional die casting and Dual-Control Forming (DCF) indicate that DCF virtually eliminates prevalent internal shrinkage cavities by intensifying feeding pressure. Consequently, the ultimate tensile strength (UTS) is elevated from 149.4 MPa to 207.0 MPa, accompanied by a remarkable surge in fracture elongation from 5.6% to 10.0% [38]. Such performance enhancements not only optimize the fracture mode but also provide crucial methodological insights for achieving high toughness and near-net-shape manufacturing of large-scale automotive wheels with complex geometries. Furthermore, alloying with RE elements has emerged as a key strategy for overcoming performance limitations in cast wheels. For instance, in the RE-containing AE81M alloy, the further micro-addition of 1% Ca promotes the formation of a dense protective film; this elevates the Weibull modulus to 4.7 in its strength distribution, significantly enhancing service reliability under complex operating conditions [39].

In the field of high-performance Mg-Nd-Zn-Zr series alloys, rheo-extrusion casting technology has demonstrated significant potential. Studies reveal that under a pressure of 80 MPa, the α-Mg grain size in Mg-2.9Nd-0.18Zn-0.35Zr wheels can be refined to 34 μm. Following T6 heat treatment, the precipitation of short rod-like β′-phase and disc-shaped β″-strengthening phase within the microstructure elevates the tensile strength to 305 MPa [40]. For Mg-2.96Nd-0.21Zn-0.39Zr wheels produced via low-pressure metal mold casting, T6 treatment increases the average fatigue strength from 68 MPa to 86 MPa. The underlying mechanism involves shifting the fatigue initiation sites from casting porosity to oxides or slip bands, which significantly extends the overall fatigue life of the alloy [41].

### 3.2. Wrought Magnesium Alloy

Through thermomechanical processing, wrought magnesium alloy wheels undergo profound grain refinement and the consolidation of internal casting defects, thereby yielding exceptionally high fatigue limits. Research in this field primarily focuses on typical grades such as AZ80 (and its RE-modified variants) and AZ31 within the Mg-Al-Zn series, as well as ZK61-Y from the Mg-Zn-Zr series.

Currently, AZ80 serves as the predominant material for the industrial-scale production of high-performance forged wheels. Its robust precipitation strengthening response imparts an exceptional strength–toughness synergy to the components [42,43]. Studies on extruded AZ80 wheels reveal pronounced microstructural heterogeneity: the rim section, having undergone intensive dynamic recrystallization (DRX), exhibits significantly refined grains down to 17.2 μm, markedly finer than the 30.5 μm grain size in the disk section. Benefiting from substantial fine-grain strengthening, the rim section exhibits outstanding mechanical properties, including a tensile strength of 339 MPa and a yield strength of 225 MPa [44]. Precise control of the process window is critical for maintaining a uniform and fine microstructure. Research indicates that excessively high ram speeds (e.g., 5 mm/s) induce grain coarsening due to severe adiabatic heating, whereas overly slow speeds (e.g., 1 mm/s) lead to similar coarsening via prolonged thermal exposure. Consequently, strictly confining the extrusion speed to an optimal window of 3–5 mm/s is paramount for preserving microstructural fidelity [45]. Another study indicates that the optimal billet temperature should be maintained at approximately 390 °C. If the forming temperature exceeds 420 °C, deformation heat readily induces rapid grain coarsening to 57.7 μm, thereby severely deteriorating the mechanical properties of the alloy [46]. Regarding modification studies, the addition of 0.4% Ce induces high-density tensile twins and effectively suppresses harmful discontinuous precipitation (DP), increasing the yield strength to 295.36 MPa while maintaining 10% elongation [47]. Furthermore, incorporating 1.5 wt.% lanthanide mixed RE elements into the AZ80 alloy enhances grain refinement capability and deformation uniformity. At 400 °C and an extrusion speed of 3 mm/s, this addition promotes a higher DRX fraction; consequently, the resulting wheel exhibits increased microhardness and a weakened basal plane texture during forming [48].

ZK61-Y is a representative grade modified with Y to enhance performance limits. Research confirms that under liquid die forging pressure, the addition of Y promotes the formation of *W*-phase and *Z*-phase, thereby refining the grain size. Experimental results indicate that at 380 °C, the combined effects of DRX and second-phase particles enable the wheel hub to achieve a tensile strength of 315 MPa and a yield strength of 150 MPa. Furthermore, following solid solution treatment, the material exhibits a remarkable fracture elongation of 25.5%, signifying an exceptional strength–ductility balance [49].

AZ31 magnesium alloy has emerged as a highly promising material for lightweight components such as automotive wheels due to its high specific strength and excellent plastic forming potential. Compared with cast magnesium alloys, wrought AZ31 alloys achieve superior comprehensive mechanical properties through subsequent thermomechanical processing. Specifically, studies demonstrate that hot spin forming at 400 °C dramatically refines the average grain size from 17.6 μm to 5.8 μm, achieving a tensile strength of 296.5 MPa and an elongation of 20.1% [50]. A study employing grey relational analysis to regulate key spinning parameters, such as axial displacement and thinning ratio, revealed that controlling these parameters effectively suppresses wheel hub wall thickness deviation (reducing it by approximately 28.84%) and forming distortion (decreasing inner diameter warpage deviation by 4.88%), providing crucial process support for the high-precision forming of magnesium alloy wheels [51]. Furthermore, regarding the hybrid cast-forge process for the AZ31 alloy, studies have systematically evaluated the regulatory effects of pouring temperature and applied pressure on microstructure, tribological properties, and corrosion resistance. Research indicates that an applied pressure of 50 MPa coupled with a pouring temperature of 700 °C yields optimal grain refinement in the wheel castings (achieving an average grain size of 11.4 μm), thereby conferring superior wear and anti-corrosion performance [52]. Additionally, a patent proposes an improved AZ31 squeeze casting process. Through the synergistic effects of a pouring temperature of 670–690 °C, a mold preheating temperature of 240–260 °C, and an applied pressure of 60–100 MPa, the micro-addition of 0.2–0.4 wt.% Ti acts as an effective grain refiner (reducing grain size from 8–15 μm to 6–10 μm), resulting in a wheel tensile strength exceeding 340 MPa (significantly higher than the 290 MPa of conventional AZ31 alloy) while being devoid of macroscopic internal defects [53].

In summary, the ultimate performance of magnesium alloy wheels depends on the synergistic interaction between alloy composition and forming processes, both of which determine mechanical response through microstructural control. To systematically compare characteristics across different technological approaches, Table 2 summarizes core processes and key performance data for representative magnesium alloy wheel materials.

## 4. Forming Technologies for Magnesium Alloy Automotive Wheels

**Figure 3:**
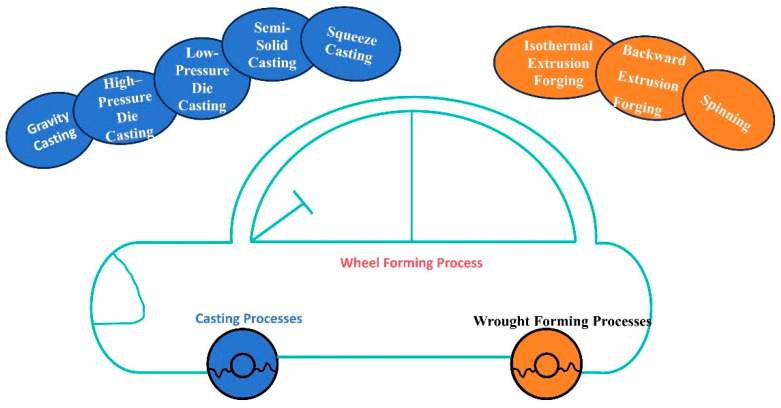
Forming processes for magnesium alloy wheels.

The manufacturing routes for magnesium alloy automotive wheels are broadly categorized into casting and wrought forming processes, as illustrated in Figure 3. Casting processes (such as gravity casting, low-pressure die casting, high-pressure die casting, semi-solid casting, and squeeze casting) focus on achieving the near-net-shape production of complex geometries through the precise control of mold filling and solidification. Conversely, wrought forming processes (such as forging and spinning) rely on solid-state plastic deformation to significantly refine the microstructure, consolidate internal casting defects, and optimize grain flow, thereby substantially elevating the overall mechanical performance of the components. Furthermore, hybrid forming processes that integrate the advantages of both approaches are increasingly becoming a key direction in the manufacturing of high-performance wheels.

### 4.1. Casting Processes

Casting is the core process for manufacturing magnesium alloy wheels, fundamentally governed by the physical metallurgy of the liquid-to-solid phase transformation. This process entails introducing molten metal into a pre-fabricated mold cavity, followed by controlled cooling and solidification to yield a cast preform. Its key advantage lies in enabling the cost-effective, integral manufacturing of wheels featuring complex geometries.

#### 4.1.1. Gravity Casting

**Figure 4:**
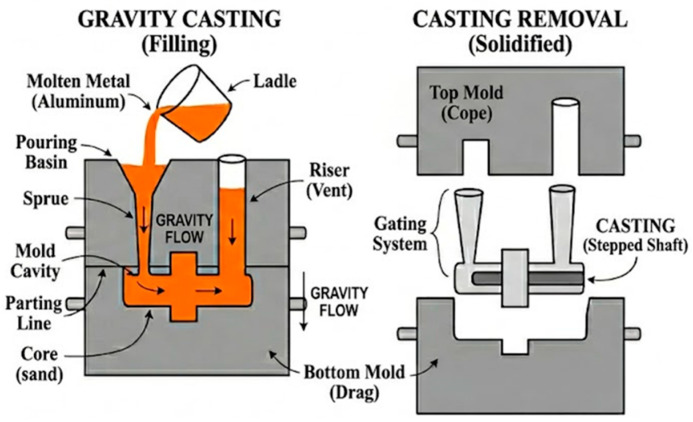
Schematic structure of gravity die casting mold for magnesium alloy wheels.

Gravity casting is a traditional method for manufacturing magnesium alloy wheels, fundamentally relying on the self-weight of the molten alloy to fill the mold cavity. The process typically employs a bottom-pouring system configured with a waist-shaped riser to enhance shrinkage compensation, offering advantages such as a simplified process flow and low capital investment [55]. Figure 4 illustrates the typical structure of a gravity die casting mold. Unlike high-pressure casting methods, this technique relies entirely on the natural gravitational force to fill the sprue, runner, and mold cavity, eliminating the need for external pressurization. The schematic details the distinct stages: mold closing, pouring the molten charge from a ladle, and finally, separating the mold halves (cope and drag) to retrieve the solidified casting and its attached gating system. However, due to the inherent susceptibility of magnesium melts to oxidation and their unstable mold-filling dynamics, gravity-cast wheels are prone to casting defects—notably hot tearing and shrinkage porosity—within geometrically complex, thin-walled sections such as rims and spokes. This limits their service reliability in high-performance applications [56,57]. To mitigate these intrinsic limitations and elevate the structural integrity of gravity-cast magnesium alloy wheels, contemporary research efforts are primarily directed toward three interconnected strategies: advanced alloy design, casting process optimization, and post-solidification thermal treatments. The addition of rare-earth elements is a key method for improving the as-cast microstructure. Zhu et al. [58] demonstrated that alloying AZ91 with 0–1.6 wt.% La promotes the precipitation of a novel Al_11_La_3_ phase. This phase effectively fragments the interconnected, network-like β-Mg_17_Al_12_ phase and significantly refines the grain size. An optimal mechanical response was realized at a La content of 0.4 wt.%, where the yield strength, tensile strength, and elongation reached 103.5 MPa, 169.75 MPa, and 4.4%, respectively.

Regarding casting process improvements, research has focused on directly controlling defects by optimizing forming process parameters. Cai et al. [55] successfully mitigated hot tearing and shrinkage porosity within the spoke and rim regions of AM60B magnesium alloy wheels during trial production. This was achieved through the synergistic tailoring of the bottom-pouring gating system’s cross-sectional ratio, the feeding efficiency of the risers, die thermal management, and the core-extraction timing. The optimized castings exhibited a 90% yield in hermeticity evaluations and withstood demanding mechanical validations, specifically a 13,730 N load radial fatigue test (10,000 revolutions) and a 230 mm drop height impact test.

To systematically enhance the performance of magnesium alloy wheels, recent studies have emphasized the synergistic optimization of alloy design, forming processes, and heat treatment regimes. Lin [57] employed a microalloying design based on the AZ91 alloy by adding elements such as Sb, Sr, and Ce, thereby developing the AE81 alloy (Mg-8Al-0.7Zn-0.25Mn-0.25Sb-0.1Sr-1.5Ce). By combining metal mold gravity casting with a heat treatment schedule of solution treatment at 410 °C for 10 h followed by aging at 200 °C for 12 h, synergistic control over the alloy’s microstructure and mechanical properties was achieved. The microstructure consists of α-Mg matrix, Mg_17_Al_12_ phase, and microalloyed phases including Al_11_Ce_3_ and Al_10_Ce_2_Mn_7_. After solution treatment at 410 °C for 10 h, in which the Mg_17_Al_12_ phase is significantly dissolved back into the matrix, while the rare-earth-containing phases remain stable. Following optimization, the AE81 alloy in the solution-treated condition exhibited a room-temperature UTS of 275 MPa, a YS of 132 MPa, and an elongation of 16.5%.

#### 4.1.2. High-Pressure Die Casting (HPDC)

High-pressure die casting (HPDC) is a pivotal technique for magnesium alloy wheel manufacturing. Its core mechanism—high-pressure, high-velocity filling coupled with rapid solidification—enables high efficiency, dimensional accuracy, and excellent surface finish, facilitated by precisely designed gating. However, the inherent characteristics of this process also introduce critical limitations: the highly turbulent, high-velocity filling readily induces gas entrapment and oxide inclusions, leading to internal gas defects. Concurrently, inadequate feeding capability can cause shrinkage cavities and porosity, while the severe processing conditions compromise die life. Zhang et al. [59] established a critical process window for achieving a dense microstructure in AM60B magnesium alloy wheels via multi-parameter optimization. The optimized parameters included a pouring temperature controlled at 680–700 °C, a die preheating temperature of 240–280 °C, a specific injection pressure of 180–200 MPa, and a holding time of 20–25 s. Experiments confirmed that wheels produced under these parameters achieved a UTS of 218–227 MPa and an elongation of 9.8–10.7%, preliminarily meeting the technical performance standards for automotive wheels.

**Figure 5:**
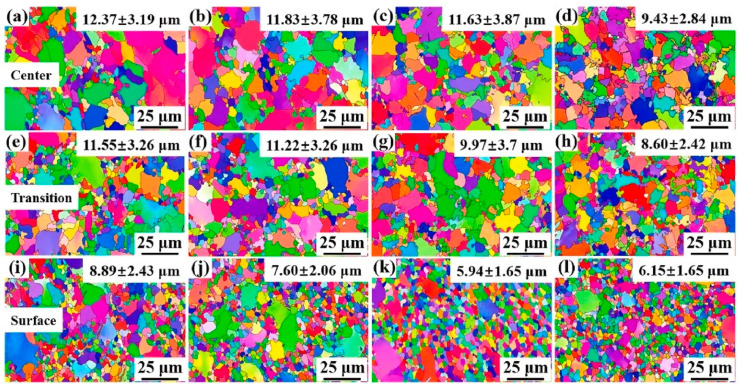
EBSD microstructure under various intensification casting pressure conditions (Different colors represent different grain orientations): (**a**,**e**,**i**) 15 MPa, (**b**,**f**,**j**) 20 MPa, (**c**,**g**,**k**) 25 MPa, (**d**,**h**,**l**) 30 MPa [60].

**Figure 6:**
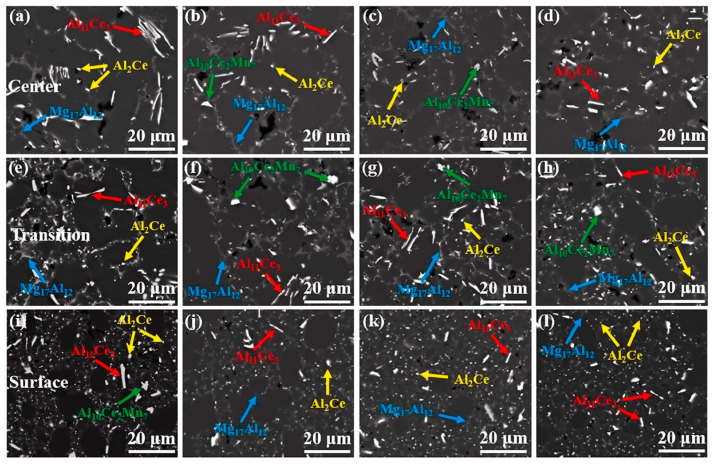
BSE-SEM microstructure under various intensification casting pressure conditions: (**a**,**e**,**i**) 15 MPa, (**b**,**f**,**j**) 20 MPa, (**c**,**g**,**k**) 25 MPa, (**d**,**h**,**l**) 30 MPa [60].

Xie et al. [60] investigated the effect of intensification casting pressure on the microstructure, defect distribution, and mechanical properties of high-pressure die casting (HPDC) AE81 magnesium alloy. Figure 5 reveals a bimodal EBSD microstructure across different intensification pressures. Coarse grains (ESCs) originate in the chamber and fragment under high shear stress during high-speed filling. In contrast, fine primary α-Mg grains form via rapid solidification in the mold cavity. Spatially, grain size decreases from the center to the surface due to a higher temperature gradient at the mold interface. Furthermore, increasing intensification pressure significantly refines the microstructure, reducing the average grain size from 10.94 μm at 15 MPa to a minimum of 8.06 μm at 30 MPa. As shown in the BSE-SEM microstructures (Figure 6), the alloy under different intensification pressures mainly consists of an equiaxed α-Mg matrix and various intermetallic compounds, including gray mesh-like Mg_17_Al_12_, bright white blocky Al_10_Ce_2_Mn_7_, bright white needle-like Al_11_Ce_3_, and particulate Al_2_Ce. From the core to the surface region of the sample, the size of these second phases gradually decreases; concurrently, the bright white phases transition from blocky to needle-like and particulate forms, while the gray phase shifts from a continuous mesh to a discontinuous morphology. When an intensification casting pressure of 30 MPa is applied, the alloy exhibits excellent mechanical properties: the yield strength (YS) reaches 186 ± 3.5 MPa, the ultimate tensile strength (UTS) is 279 ± 2.3 MPa, and the elongation (EL) is 9.6 ± 0.8%.

**Figure 7:**
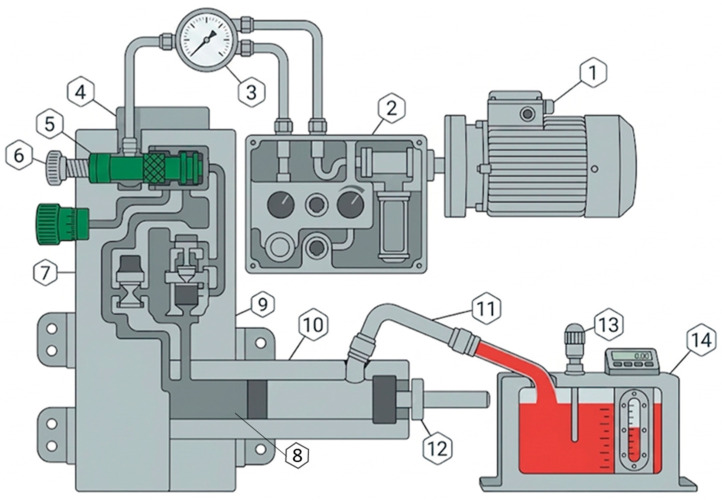
Working principle of the novel V-HPDC process. 1—Vacuum pump; 2—Negative pressure tank; 3—External vacuum valve; 4—Vacuum block; 5—Plunger; 6—Hydraulic cylinder; 7—Moving die; 8—Cavity; 9—Fixed die; 10—Shot sleeve; 11—Feeding tube; 12—Plunger tip; 13—Pouring pump; 14—Melting furnace.

Nevertheless, traditional HPDC remains unable to completely eliminate the risk of trapped gas. Consequently, vacuum high-pressure die casting (V-HPDC) has emerged as the mainstream solution for further enhancing performance. To overcome the core bottlenecks of traditional HPDC, systematic process innovations focusing on vacuum HPDC (V-HPDC) have been developed. Figure 7 shows the schematic of V-HPDC technique, utilizing cavity evacuation to minimize gas entrapment. This approach innovatively combined bidirectional pressure compensation (piston pressure retention with plunger tracking compensation) to deliver sustained, balanced solidification pressure. Coupled with sealed pouring to prevent melt oxidation, this approach synergistically optimized both filling and shrinkage compensation. This integrated strategy significantly minimized macroscopic defects and refined the microstructure of AZ711 magnesium alloy castings. Performance testing demonstrated that UTS (180–300 MPa) and specific strength (118–172 MPa) increased by over 25% and 30%, respectively, compared with conventional parts, primarily due to enhanced density. Simultaneously, the vibration damping coefficient surged by over 100% (reaching 30–60%), which is closely linked to the enhanced damping dissipation mechanism from grain refinement strengthening. This research confirms that actively controlling the filling environment and solidification pressure is an effective approach to regulating microstructure and enhancing the performance of magnesium alloy wheels. Li et al. [61] developed an integrated V-HPDC rapid injection molding process. This approach achieved comprehensive defect control throughout the entire process—from melt handling to solidification—by actively evacuating the die cavity (10 kPa vacuum), optimizing injection and holding pressure parameters (50 MPa intensification pressure, 12 s holding time), and incorporating SF_6_/N_2_ mixed gas protection. This process yields a dense and refined microstructure for AZ91D alloy, significantly enhancing its comprehensive properties: UTS reached 253.19 MPa with an EL of 18.74%. Notably, the corrosion current density was substantially reduced from 158.5 $\;\mu A/{cm}^{2}$ in conventional HPDC parts to 63.1 $\mu A/{cm}^{2}$, attributed to the synergistic effect of the fine-grained microstructure and the corrosion barrier provided by the β-Mg_17_Al_12_ phase.

Collectively, these studies demonstrate that V-HPDC can mitigate defect formation and tailor the microstructural evolution in magnesium alloys by actively controlling the filling environment and solidification pressure. Consequently, it represents a pivotal processing strategy for enhancing their overall properties. Furthermore, the systematic investigations into various process parameters—such as tailored pressure intensification methods, optimized injection velocities, and specialized protective atmospheres—offer versatile and viable technical pathways for defect mitigation and performance optimization in magnesium alloy wheels.

#### 4.1.3. Low-Pressure Casting (LPC)

**Figure 8:**
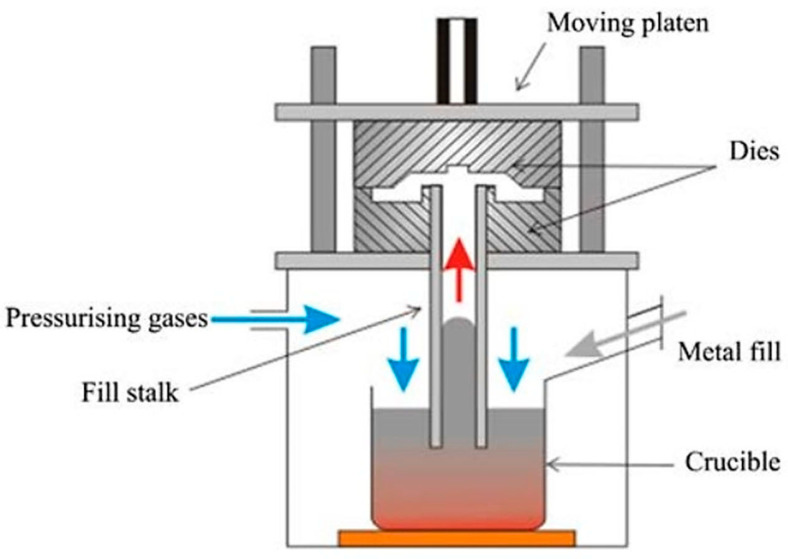
Schematic of the LPC machine for magnesium alloy wheels [64].

Low-pressure casting (LPC) is a precision forming process that achieves smooth filling and directional solidification by applying an external pressure (typically 0.2–0.5 MPa) during the solidification phase of the melt [62]. Its technological origins trace back to the 1906 patent by Wetherill et al. [63], which laid the foundation for modern equipment. The core principle involves introducing a dry inert gas onto the surface of the molten metal within a sealed crucible. Driven by this pressure, the melt ascends smoothly through the riser tube, filling the mold cavity (as illustrated in Figure 8 [64]). Directional solidification then proceeds under sustained pressure. Upon completion of solidification, the pressure is released, allowing the unsolidified metal in the gating system to flow back. This process not only effectively reduces the risks of turbulent filling and gas entrapment but also unifies the filling and feeding channels. This fundamentally harmonizes the temperature and pressure fields within the mold, resolving the contradiction between smooth filling and efficient feeding that is inherent in traditional gravity casting.

**Figure 9:**
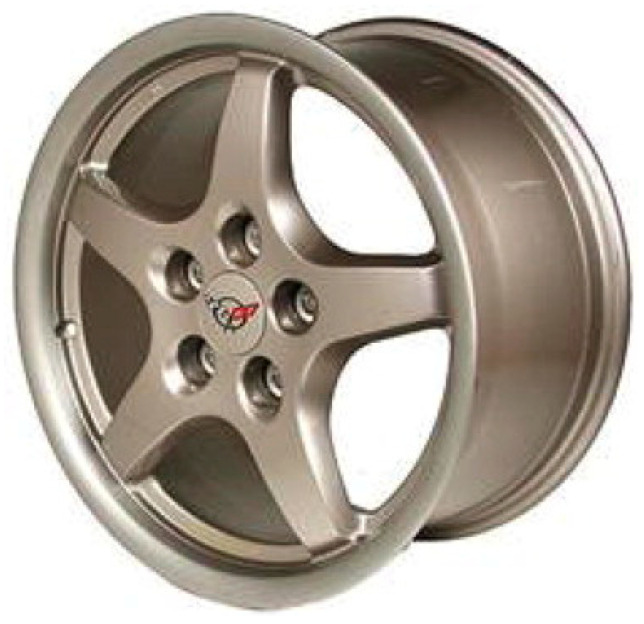
LPC magnesium alloy wheel for the Chevrolet Corvette [65].

As a core technology for manufacturing structurally complex magnesium alloy wheels with outstanding mechanical properties, LPC has demonstrated significant advantages in the high-performance vehicle sector. A representative industrial benchmark is the magnesium alloy wheels developed for the Chevrolet Corvette (Figure 9) [65], which compellingly underscores the lightweighting potential of the LPC process for high-performance automotive chassis components. Despite these successful applications, the precise control of internal defects remains a primary challenge for LPC magnesium alloy wheels. Currently, research efforts are primarily focused on two levels: macroscopic process parameter optimization and localized solidification regulation.

In the field of process optimization, systematic investigations have focused on tailoring key parameters. For instance, targeting AZ91D magnesium alloy wheels, Chen et al. [34] employed multi-objective optimization using the SiPESC platform combined with the Ant Colony Optimization (ACO) algorithm. They determined the optimal process parameter combination to be a pouring temperature of 689 °C and a filling pressure of 6.5 kPa. This significantly reduced the internal shrinkage porosity rate from 4.1% to 2.1% and refined the secondary dendrite arm spacing (SDAS) from 88.5 μm to 81.2 μm. Regarding physical design and performance validation, Kim [66] proposed a “volumetric effect compensation” strategy for 18-inch AZ91D wheels. Increasing the rim wall thickness from 4/3/4 mm in the aluminum alloy prototype to 5/5/5 mm effectively compensated for the inherent strength disparity between the materials. Experimental results confirmed that the wheel produced via LPC followed by a T4 heat treatment weighed 8.5 kg, achieving a substantial 26.0% weight reduction compared with the aluminum alloy prototype. Mechanical characterization revealed a measured UTS of 224.8 MPa and an EL of approximately 7.8%, outperforming comparable commercial products and meeting fundamental performance standards. However, constrained by melt impurities, the YS (approximately 133.7 MPa) still has room for improvement and requires further enhancement through subsequent heat treatment processes.

**Figure 10:**
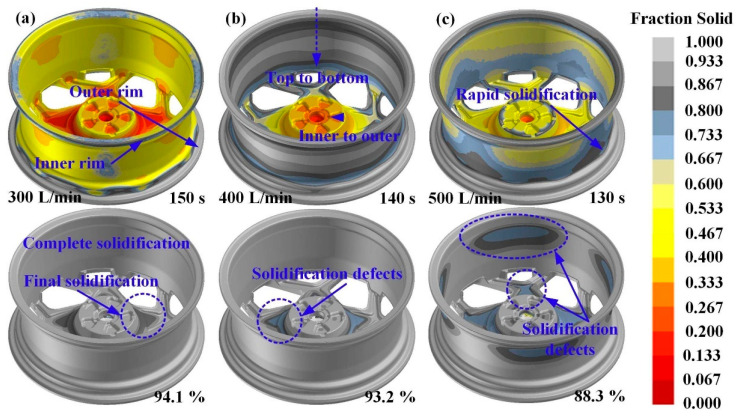
Solidification process of the LPDC aluminum alloy wheel hub under different cooling processes: (**a**) 300 L/h for 150 s; (**b**) 400 L/h for 140 s; (**c**) 500 L/h for 130 s [68].

For wheels characterized by complex geometric features, the formation and elimination of internal defects—such as shrinkage cavities in the hub region—often cannot be achieved through global process parameter adjustments alone. The fundamental solution to such defects relies more on the active regulation of the local solidification sequence. Taking AZ91D magnesium alloy wheels as an example, optimizing parameters like pouring temperature and filling rate was inadequate; even within reasonable processing windows, shrinkage cavities remained unavoidable because the hub region consistently served as the final zone to solidify. Consequently, the study shifted focus to the structural optimization of the mold cooling system. Ying et al. [67] found that by employing simulation calculations, they could effectively predict the temperature field distribution during solidification and evaluate various cooling system designs. The simulation assisted in establishing ideal directional solidification conditions, which ultimately resolved the issue of shrinkage cavities and optimized the overall solidification sequence. Furthermore, simulation calculations can also be utilized to optimize cooling process parameters. The LPDC process of the wheel hub was numerically simulated by ProCAST software to investigate forming quality under different cooling strategies (Figure 10) [68]. The results indicated that the melt exhibited different solidification characteristics under three specific cooling processes: 300 L/h for 150 s, 400 L/h for 140 s, and 500 L/h for 130 s. An optimal sequential solidification state was obtained under the condition of 400 L/h for 140 s. By comparing the tensile properties of the rim, it was found that wheel hubs cast under this specific condition exhibited higher tensile strength and elongation.

In summary, research on LPC process optimization exhibits a distinct evolutionary trajectory: transitioning from the global optimization of macroscopic process parameters (e.g., pressure and temperature) to the precise tailoring of localized solidification behavior in geometrically intricate components through structural design modifications, such as optimizing die cooling conditions.

#### 4.1.4. Semi-Solid Casting (SSC)

**Figure 11:**
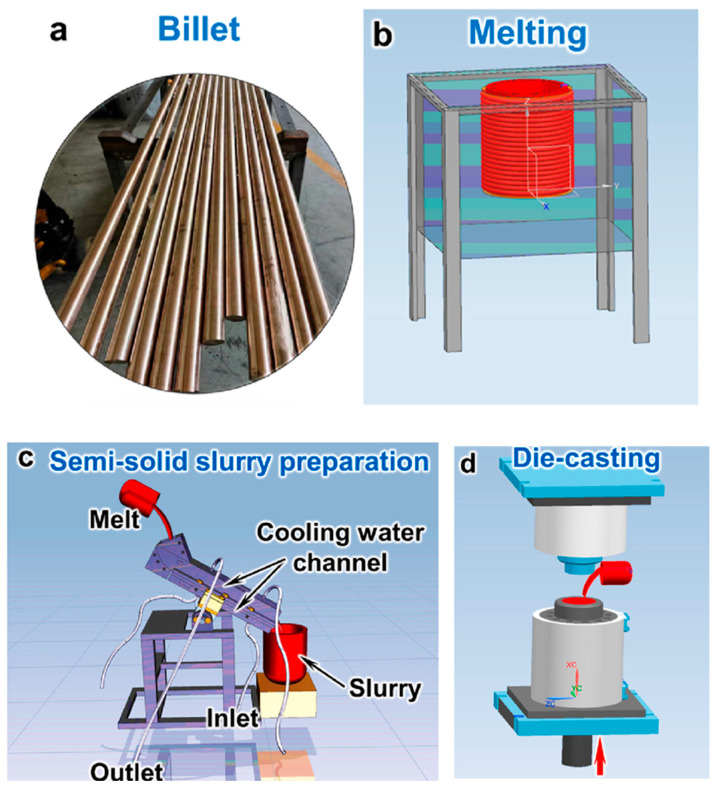
Schematic of the SSC mold: (**a**) Billet; (**b**) Melting; (**c**) Preparation of semi-solid slurry; (**d**) Squeeze casting [69].

Semi-solid casting (SSC) is an advanced near-net-shape forming process that employs a metal slurry within the solid–liquid two-phase region. The key to this process lies in the precise control of the formation and evolution of non-dendritic microstructures, facilitating the exact manufacturing of geometrically intricate components via a stable, low-velocity filling process. This technique effectively mitigates defects such as gas entrapment and inclusions typically induced by turbulent flow in conventional liquid casting. Furthermore, because the solidification shrinkage of semi-solid materials is inherently reduced, it significantly alleviates thermal stresses within the castings and the mechanical load on the dies, offering an innovative solution for manufacturing high-performance lightweight components. As illustrated in Figure 11 [69], the SSC process integrates melting, slurry preparation, and squeeze casting. Following complete melting and slag removal, the molten alloy is cooled to a predefined temperature and poured through a cooling slope device to generate a semi-solid slurry containing a primary solid phase. The prepared slurry is then collected, held isothermally for a brief duration, and poured into a preheated mold where squeeze casting is immediately performed under pressure.

SSC is widely recognized as a key process for mitigating turbulence-induced defects prevalent in conventional casting, as it leverages the unique rheological properties of semi-solid slurries to achieve stable die filling. The successful implementation of SSC relies heavily on robust numerical simulations, the reliability of which stems from the accurate constitutive modeling of the material’s rheological behavior. For instance, Li [70] developed a constitutive model for the AZ91D magnesium alloy that couples temperature, strain rate, and solid fraction, providing critical inputs for subsequent simulations. Numerical simulations based on such models further indicate that for the thixoforging of magnesium alloy wheel hubs, the billet temperature (565–575 °C) and punch velocity (1.5–2.5 m/s) form a synergistic core process window for controlling fluidity and preventing folding defects. Concurrently, mold radius optimization plays a decisive role in enhancing metal flow uniformity. Comprehensive studies demonstrate that through these systematic optimizations, SSC can effectively refine the microstructural features (e.g., globular particle size) of magnesium alloys to 50–80 μm and elevate the UTS to 220–240 MPa, delivering significantly superior performance compared with conventional die casting.

Focusing on the precise optimization of slurry preparation processes, Gan et al. [71] conducted systematic experimental studies on the AZ91D magnesium alloy. Through designed comparative experiments (fixed temperature of 655 ± 5 °C, adjusting holding time and casting pull rate), they clearly isolated and quantified the influence of each process parameter. The study revealed that casting pull rates varying between 13–21 cm/min only marginally improved microstructure and properties. In stark contrast, holding time exhibited a pivotal regulatory role: within the 20–40 min range, increasing holding time led to significantly rounded and refined primary α-Mg grains, while coarse, irregular Mg_17_Al_12_ precipitates gradually dissolved and reprecipitated as fine, dispersed phases, resulting in a marked increase in TS. However, for holding times exceeding 40 min, the microstructure stabilized, and the mechanical properties experienced a slight decrement. This evolutionary trajectory precisely elucidates the mechanisms underlying the microstructural optimization of semi-solid slurries via the precise control of isothermal holding time. Under the optimized processing conditions, the TS of specimens reached 299.8 MPa, fully demonstrating that significant improvements in the mechanical properties of semi-solid AZ91D magnesium alloy can be achieved through rigorous process parameter optimization.

#### 4.1.5. Squeeze Casting (SC)

**Figure 12:**
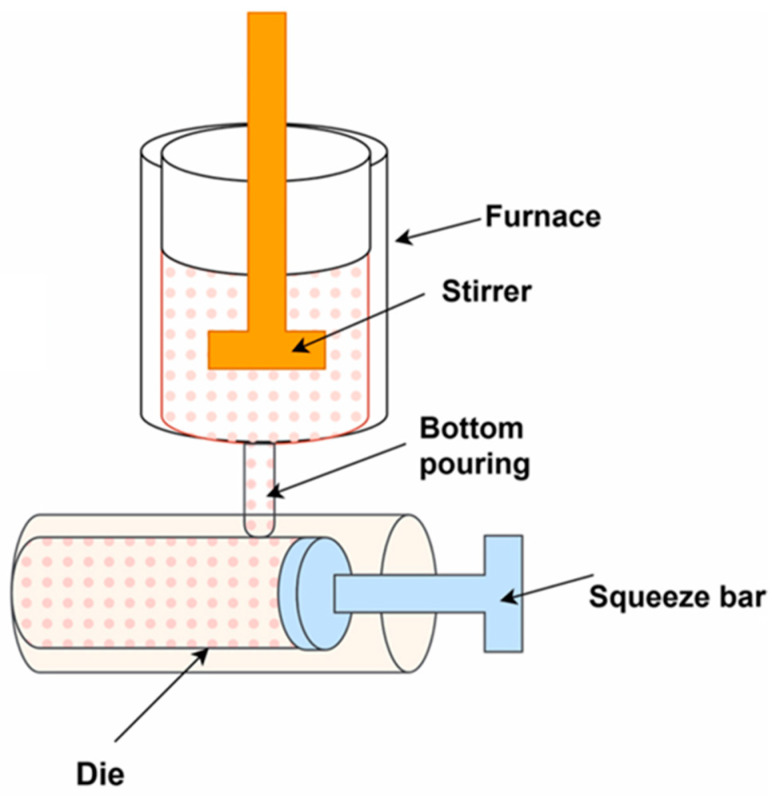
Schematic representation of the working procedures in the indirect squeeze casting process [73].

Squeeze casting (SC) is a near-net-shape forming process that synergizes liquid metal filling with solidification under high pressure [72]. Its core process consists of four stages: pouring, mold closing and filling, pressure solidification and demolding (Figure 12) [73]. Its principle involves applying sustained high mechanical pressure to the partially solidified metal via the die, thereby driving crystallization under a highly compressive state [74]. Because the pressure acts directly on the solidification front, it forces the melt to feed microscopic voids induced by solidification shrinkage. This fundamentally eliminates inherent defects prevalent in magnesium alloys—such as shrinkage cavities and porosity—that typically arise from their wide freezing ranges (crystallization intervals). Consequently, this technology integrates the shape-casting versatility to form geometrically intricate components with the advantages of forging processes in enhancing microstructural densification.

In actual manufacturing environments, to mitigate the severe oxidation susceptibility of magnesium alloys and the inherent fluctuations in process parameters, Xu et al. [75] proposed an integrated novel SC approach. This process achieves stable mold filling devoid of atmospheric interference by directly drawing the melt from a sealed crucible through a riser preheated to 400 °C–550 °C. An automated pressure compensation system ensures precise and constant pressure throughout solidification. As a representative application, the trial production of LX150 motorcycle magnesium alloy wheels demonstrated a 20% reduction in cycle time concomitant with significant improvements in casting yield and densification. This provides crucial processing guidelines and technological reserves for the economical production of large-scale, high-performance automotive magnesium alloy wheels. To further tailor component quality, Yao et al. [76] conducted optimization studies using a VSC-1500 SC machine based on an *L_b_* (4^1^ × 2^4^) mixed-level orthogonal experimental design. Simulations revealed that shrinkage cavities preferentially nucleate and cluster at the hub center and the rim–spoke junctions. The study identified the optimal parameter combination: a filling velocity of 600 mm/s, an extrusion pressure of 130 MPa, a mold temperature of 200 °C, and a pouring temperature of 690 °C. Results indicate that applying high pressure significantly mitigates microscopic shrinkage porosity and enhances casting density. Furthermore, Lin et al. [77] employed the finite element method to establish a model comprising approximately 970,000 high-precision volume elements, enabling the rigorous quantification of the thermo-mechanical coupled behavior in wheel hub SC. The simulation revealed a stepwise evolution of the temperature field from the rim to the hub during the filling stage and established the sensitivities of defect suppression to die temperature and extrusion pressure. This provides a quantitative theoretical basis for ensuring microstructural densification via the precise feedback regulation of pressure parameters.

### 4.2. Wrought Forming Processes

Wrought forming represents a pivotal processing route for enhancing the overall properties of magnesium alloy wheels [78]. Its fundamental mechanism relies on exploiting the plastic flow capacity of solid metals. By applying compressive stresses via dies at elevated temperatures, the billet (or blank) undergoes macroscopic permanent deformation to achieve the desired geometry. Unlike casting, this process fundamentally circumvents liquid–solid phase transitions. Instead, it facilitates profound grain refinement and optimizes macroscopic flow lines through dynamic/static recrystallization (DRX/SRX) mechanisms, thereby simultaneously achieving the synergistic tailoring of both component geometry and intrinsic mechanical properties.

#### 4.2.1. Isothermal Extrusion Forging

Isothermal extrusion forging is a process wherein both the billet and the die are maintained at an identical, constant temperature, forming components through a coupled extrusion–forging action. Its core advantage lies in effectively suppressing microstructural inhomogeneity caused by temperature fluctuations. Deforming the material at low strain rates under these isothermal conditions significantly reduces forming loads and enhances dimensional accuracy, making it particularly suitable for manufacturing geometrically intricate components from materials with inherently limited ductility, such as magnesium alloys [79]. However, this process remains constrained by limitations such as prolonged production cycles and stringent requirements for equipment temperature control precision, which limit its large-scale industrial application.

**Figure 13:**
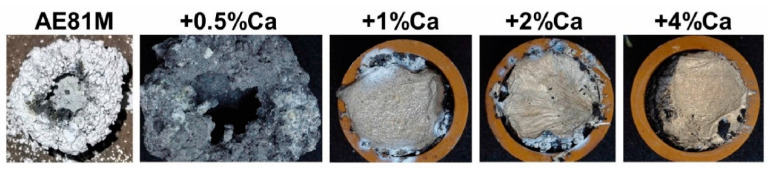
Samples resulting from the experiment that investigates the melt oxidation process of AE81M-xCa alloy in an atmosphere of air [39].

In the study of isothermal extrusion forging for magnesium alloy wheels, elucidating the mechanistic correlations between processing parameters and the resulting microstructural and mechanical responses represents a critical focus. Zhao et al. [44] employed a homogenization pretreatment coupled with an isothermal, low-velocity (1 mm/s) forming protocol, which effectively mitigated macroscopic cracking. A pivotal finding was that severe plastic deformation continuously drives DRX under isothermal conditions. Due to uneven strain distribution, the degree of recrystallization exhibits significant regional variations. The rim section, subjected to higher levels of plastic strain, exhibited an average grain size refined to approximately 17.2 μm, accompanied by a weakened crystallographic texture. In contrast, the hub section (wheel disk), experiencing less deformation, showed an average grain size of about 30.5 μm. This microstructural differentiation directly resulted in a gradient distribution of mechanical properties: the rim section exhibited significantly superior UTS (339 MPa) and elongation at break (14.6%) compared with the hub section. Additionally, the high density of grain boundaries in the highly deformed region substantially enhanced its fatigue performance. To evaluate the consistency of mechanical properties in cast wheels, Li et al. [39] utilized the Weibull modulus while analyzing melt oxidation during mold filling. They discovered that atmospheric conditions dictate the effectiveness of calcium addition in the AE81M alloy. Under standard air, calcium effectively curbs severe burning and oxide defects (Figure 13).

To overcome the engineering challenges of low manufacturing efficiency and prohibitive load requirements, Wang et al. [80] proposed an innovative solution based on an isothermal extrusion forging strategy utilizing hollow billets. Using AZ80 alloy as the target material, the process employs homogenized hollow billets to manufacture monolithic wheels through a three-step near-net-shape process—reverse extrusion, pre-forging of the lip, and expansion forming—at a constant temperature of 320–380 °C. The hollow billet design substantially reduced the contact area and interfacial stress, significantly lowering forming loads. Manufacturing was achieved using only a 12.5 MN press while minimizing subsequent machining steps. The resulting wheels exhibit full filling, high precision, and easy demolding, achieving a 28% weight reduction. More importantly, this process imparts a greater magnitude of plastic strain throughout the component, which profoundly stimulates DRX and consequently fortifies the mechanical properties across all wheel sections. The UTS reaches 300–320 MPa, with an elongation to failure exceeding 10%.

In summary, isothermal extrusion forging exhibits distinct advantages in enhancing the microstructural integrity and mechanical properties of magnesium alloy wheels. Current research encompasses both fundamental mechanistic investigations (e.g., Zhao et al.’s analysis of microstructural evolution patterns [44]) and applied engineering optimization (e.g., Wang et al.’s process innovations aimed at reducing load and improving efficiency [80]). This reflects the parallel progression of fundamental materials science and technological deployment in this field. Future endeavors should principally focus on optimizing the synergistic processing windows of key parameters, particularly temperature and strain rate. This strategy aims to preserve the material’s intrinsic microstructural and mechanical merits while further augmenting process efficiency and economic viability, thereby accelerating its widespread industrial implementation.

#### 4.2.2. Backward Extrusion Forging

**Figure 14:**
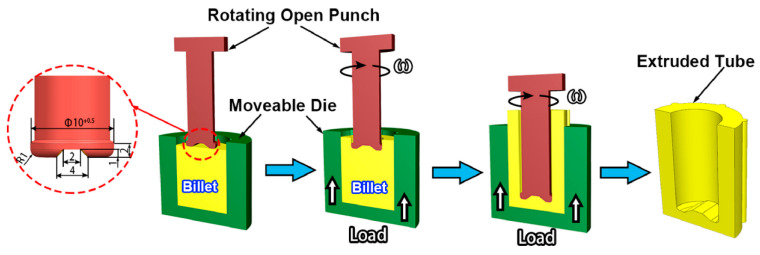
Schematic diagram of the backward extrusion process using a hollow billet [82].

Backward extrusion is a plastic forming process that drives solid metal billets to flow in the reverse direction relative to the punch stroke, thereby filling the die cavity [81]. Compared with traditional casting, this process effectively refines grain size through intense plastic deformation, significantly enhancing the comprehensive mechanical properties of components. Compared with other forging processes, it offers superior forming capability and material utilization for manufacturing geometrically intricate components featuring deep cavities, such as wheels. The typical tooling configuration and die assembly governing this backward extrusion process are schematically demonstrated in Figure 14 [82].

**Figure 15:**
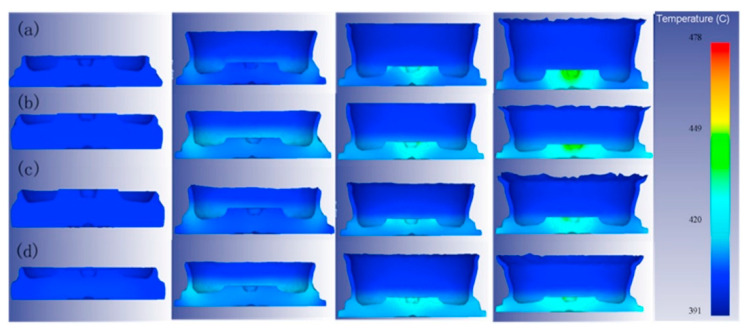
Effect of spoke thickness increment Δh on temperature field in wheel forming process: (**a**) +0 mm, (**b**) +1 mm, (**c**) +2 mm, (**d**) +3 mm [83].

**Figure 16:**
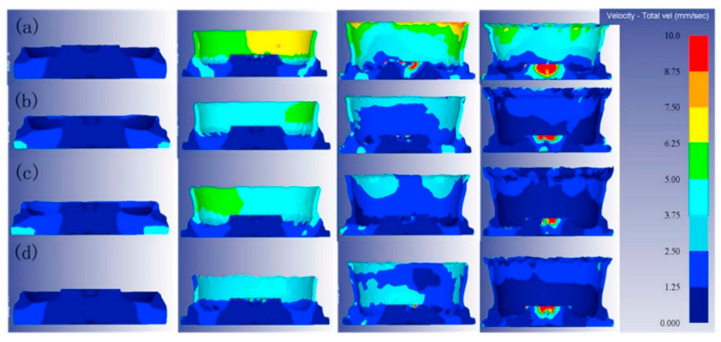
Effect of punch fillet increment ΔR on velocity field in wheel forming process: (**a**) 0 mm, (**b**) +2 mm, (**c**) +5 mm, (**d**) +8 mm [83].

**Figure 17:**
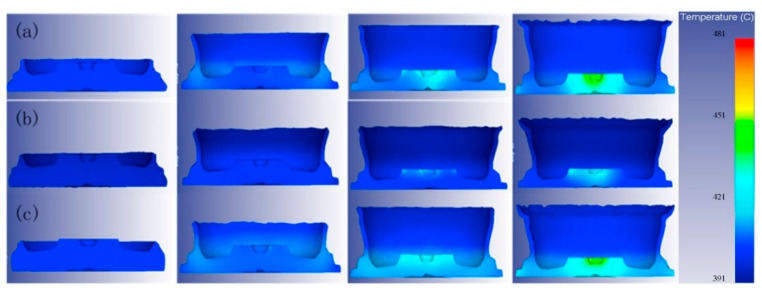
Effect of upper rim fillet increment ΔR3 on temperature field in wheel forming process: (**a**) +0 mm, (**b**) +2 mm, (**c**) +4 mm [83].

The systematic optimization of the die structure is foundational to modulating thermo-mechanical fields and mitigating defects. Research indicates that synergistically adjusting spoke geometry and critical fillet radii can effectively guide material flow while improving stress distribution and heat dissipation characteristics. For instance, Jiang et al. [83] revealed that increasing spoke thickness suppresses turbulent metal flow at the wheel base, significantly reducing the local peak temperature from approximately 450 °C to below 420 °C (Figure 15). Concurrently, properly enlarging the fillet radius of the punch can decrease flow velocity fluctuation and stress concentration, and optimize the metal flow path (Figure 16). Appropriately increasing the inner fillet radius of the upper rim relieves the shear stress at the edge and inhibits tearing defects (Figure 17). Such structural optimizations establish highly uniform boundary conditions, facilitating the precise regulation of subsequent processing parameters. Furthermore, Liao et al. [84] noted that the eccentric placement of billets during production is a primary cause of premature die failure and elevated component scrap rates. Their analysis revealed that eccentric extrusion leads to uneven rim filling heights and generates asymmetric thermo-mechanical fields within the die, resulting in severe microstructural heterogeneity. To address stress concentration and brittle fracture at the contact interface between the female and base dies—common issues in composite dies—this study proposes an optimization strategy: machining the female die contact surface into a curved surface along the fracture plane and adding a truncated cone (conical taper) to mate with the base die. This structural modification effectively eliminates harmful moments, significantly extending the fatigue life of the die while maintaining forming quality.

Based on the optimized dies, the rim wall thickness emerges as a critical process parameter that profoundly dictates the thermo-mechanical coupling and microstructural evolution during the forming process. Investigating AZ80 magnesium alloy wheels, Jiang et al. [85] systematically elucidated the mechanistic effects of rim wall thickness. Their findings revealed that increasing the wall thickness significantly reduces forming loads during the terminal stages of the process. Furthermore, the decelerated material flow mitigates deformation-induced heating, thereby enhancing the macroscopic uniformity of the thermal field. Microstructural characterization further revealed that when the degree of DRX remains stable, the average grain size exhibits a non-monotonic variation pattern of “refinement followed by coarsening” as wall thickness increases. Based on these findings, the study proposed the existence of an optimal process window (e.g., a rim wall thickness increment of approximately 14.3%) that maintains low forming resistance while achieving superior microstructural uniformity.

Beyond the optimization of macroscopic die geometry, the precise control of the thermal history during forming is equally critical. In the backward extrusion of magnesium alloy wheels, Jiang et al. [86] demonstrated that the uniformity of the die temperature field significantly dictates microstructural evolution. To address the susceptibility to localized overheating (overburning) prevalent in single-step forming, the study innovatively proposed a localized differential thermal management strategy for the die assembly (e.g., setting the base die temperature to 320–370 °C, lower than the 420 °C of the remaining die components). This approach significantly refined the grain structure at the hub base without increasing the risk of rim cracking, thereby substantially enhancing both the YS and elongation to failure within the core and spoke sections of the AZ80 wheel.

**Figure 18:**
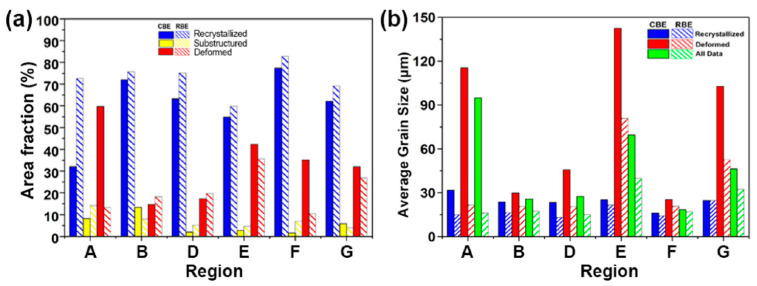
(**a**) The area fraction of recrystallization grains, sub-grains and deformed grains in different regions (A-G represent different positions on the workpiece); (**b**) The average grain size distribution of recrystallization grains and deformed grains in different regions (A-G represent different positions on the workpiece) [82].

To achieve superior microstructural control, the development of the rotating backward extrusion process through die rotation has provided a novel approach for precisely regulating material deformation and microstructure. Research by Chen et al. [87] on AZ80 magnesium alloy tubes indicates that the die motion speed ratio (λ) is the core parameter governing this process. Their key finding is that while increasing λ amplifies the radial strain gradient (with the coefficient of variation rising from 0.29 to 0.37), it significantly elevates the DRX fraction to approximately 94.8% by inducing severe shear deformation and concomitant deformation-induced heating. This microstructural optimization directly leads to the simultaneous enhancement and homogenization of overall mechanical properties. Under optimal conditions (achieving a cumulative strain of approximately 5.5), the component achieved maximum strength (UTS of 295.84 MPa) at the mid-wall thickness and optimal plasticity (elongation of 14.02%) at the inner wall. Moreover, the radial coefficient of variation for UTS was drastically reduced from 0.18 in the conventional process to 0.02. This study mechanistically reveals that regulating λ can coordinate the chain reaction among the “strain field–temperature field–recrystallization behavior,” providing a quantitative theoretical basis for fabricating high-performance uniform components. Che et al. [82] demonstrated the distinct advantages of rotating backward extrusion (RBE) over conventional backward extrusion (CBE) for processing AZ80 magnesium alloys. Compared to CBE, the RBE method yields superior deformation uniformity and equivalent strain. Microstructurally, RBE drives profound grain refinement (88.60% size reduction) and dynamic recrystallization (55.30% increase), which CBE fails to achieve to the same extent (Figure 18). Ultimately, this comparative advantage results in a weaker texture and markedly higher microhardness in RBE components.

#### 4.2.3. Spin Forming

**Figure 19:**
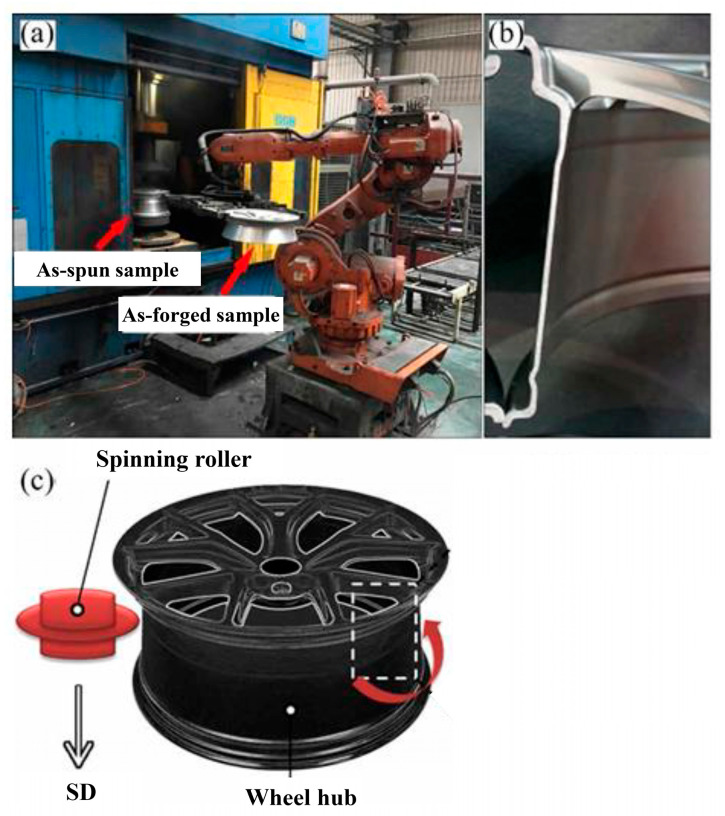
Spinning process illustrating transition from as-forged sample to as-spun sample (**a**), longitudinal view of corresponding as-spun sample (**b**), and schematic diagram during spinning process (**c**) [50].

Spin forming is a manufacturing process that applies localized, progressive plastic deformation to a rotating metal blank via rollers, ultimately conforming it to a die profile [88]. This process offers advantages in enhancing material strength, improving precision, and increasing material utilization [89]. However, when applied to complex components such as magnesium alloy wheels, challenges like uneven wall thickness distribution and difficulties in coordinating overall deformation remain prevalent. Adopting a staged spinning strategy—where the total plastic strain is strategically partitioned across successive passes with tailored control objectives for each stage—has become an effective approach to optimize material flow, modulate the stress distribution, and thereby enhance overall forming quality and performance. The schematic diagram of the spinning process is shown in Figure 19 [50].

Within the paradigm of macroscopic process design and system optimization, research focuses on improving forming uniformity by decoupling deformation objectives across successive stages. For instance, Zhang et al. [51] proposed a systematic processing strategy for AZ31 magnesium alloy wheels that fundamentally departs from conventional single-pass forming. The core of this approach involves designing a two-pass hybrid spin forming process: the first pass employs triple-wheel staggered power spinning to simultaneously achieve thinning and elongation, while the second pass uses conventional spinning to ensure final contour accuracy. This staged strategy aims to better control deformation uniformity by separating the objectives of material flow and shape conforming across distinct passes. Based on experimentally validated reliable models (prediction error < 8%) and systematic parameter optimization (orthogonal experiments combined with grey relational analysis), the study quantitatively identified axial offset as the most critical process parameter. Post-optimization, wall thickness deviation decreased by 28.8%. This work demonstrates an engineering approach to achieving quantitative process parameter design through systematic methods.

**Figure 20:**
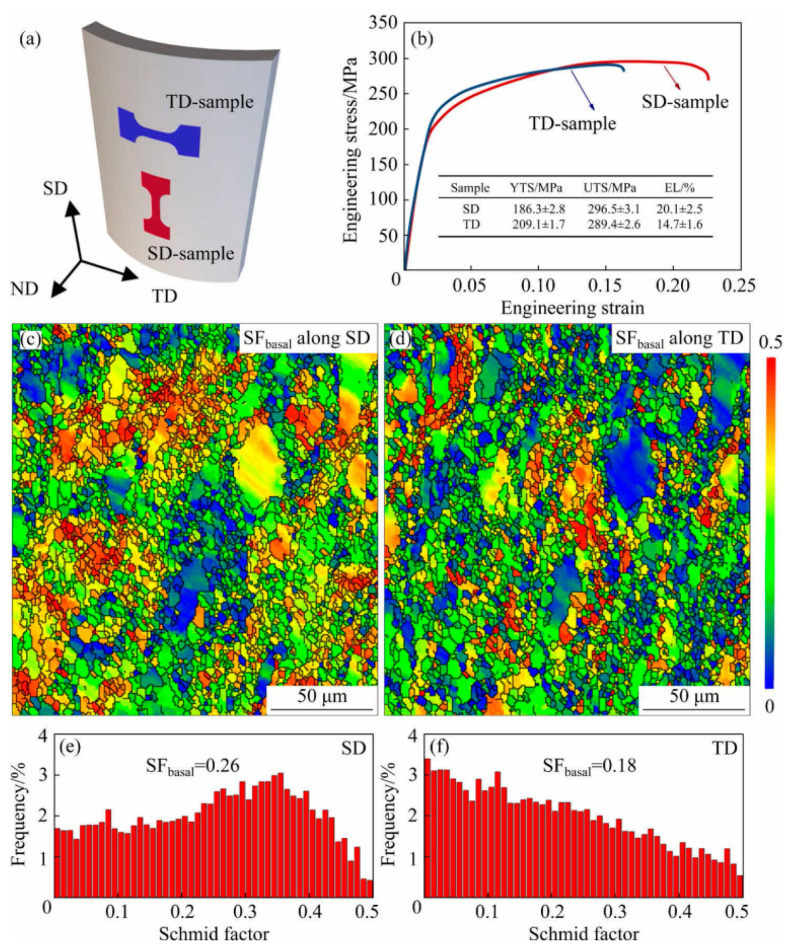
(**a**) Schematic diagram of SD- and TD-tensioned samples at Position 4 from AZ31 wheel hub; (**b**) corresponding engineering tensile stress−strain curves; (**c**−**f**) SF maps for basal slip (**c**,**d**) and corresponding average SF values (**e**,**f**) of samples tensioned along SD and TD, respectively [50].

For heavily alloyed magnesium variants exhibiting superior strength but poorer ductility, the effectiveness of a phased strategy hinges on its integration with thermal field control. Cao et al. [90] successfully addressed cracking issues during high thinning rate (50%) spinning of AZ80 magnesium alloy wheels by employing preheating to 400 °C combined with a split-pass strategy. Their systematic study revealed synergistic control mechanisms: increasing spinning temperature to 420 °C enabled complete DRX, achieving 15.8% elongation; a feed ratio of 0.10 mm/rev balanced forming stability and performance, yielding a UTS of 316 MPa. Notably, amplifying the thinning reduction from 25% to 50% induced continuous grain refinement, resulting in a remarkable 160–180% enhancement in strength relative to the as-cast state. These findings underscore that the synergistic tailoring of thermo-mechanical parameters provides a robust processing framework for the reliable manufacturing of high-strength magnesium alloys. A profound understanding of microscopic deformation mechanisms and crystallographic texture evolution provides the theoretical foundation for establishing the intrinsic relationship between process parameters and final properties. In this regard, Jiang et al. [50] systematically revealed the quantitative correlation among the “process–mechanism–texture–property” linkages during the multi-pass spin forming of the AZ31 alloy. Under the established process conditions (a spinning temperature of 400 °C, total reduction rate of 70%), the plastic deformation mechanism undergoes a fundamental shift: transitioning from initial basal plane <a> slip (accounting for ~69% of dislocation activity) to a subsequent substantial contribution from pyramidal plane <c+a> slip (accounting for ~41%). This activation transition in the slip systems is crucial for accommodating strain along the *c*-axis, thereby driving the crystal *c*-axis to tilt toward the spinning direction (SD) and forming a strong texture. This texture directly induces pronounced mechanical anisotropy (Figure 20): specimens oriented along the SD exhibit a slightly lower YS (approximately 186 MPa vs. 209 MPa) but significantly superior ductility (an elongation to failure of 20.1% vs. 14.7%). Through multiscale characterization, this work establishes a quantifiable causal chain linking micro-mechanisms to macro-properties, thereby elevating the research paradigm from phenomenological description to mechanistic insight.

In summary, multi-pass spin forming has emerged as a highly robust processing paradigm for enhancing the structural integrity and overall mechanical performance of magnesium alloy wheels. Existing research has advanced from multiple dimensions, including macro-level process system optimization, the synergistic control of thermal parameters, and the quantitative interpretation of microstructural mechanisms, thereby culminating in a comprehensive theoretical and technological framework. Future research endeavors should prioritize the development of computationally driven, intelligent process design models that integrate microstructural evolution predictions, while exploring adaptive multi-pass strategies tailored for geometrically intricate and asymmetric components. This trajectory will further enhance the scientific rigor, predictive capability, and broad industrial viability of this advanced manufacturing technology.

### 4.3. Combined Forming Processes

To circumvent the inherent limitations of conventional single-technique forming processes, hybrid forming strategies that synergize the distinct merits of multiple technologies have emerged as a pivotal trajectory in magnesium alloy wheel manufacturing [91]. Current research endeavors are predominantly directed toward synergistic innovation across two interconnected domains: macro-level forming control and microstructural regulation.

In macroscopic and defect control, researchers optimize material flow through combined deformation pathways. To overcome casting defects in the ZK61-Y magnesium alloy, Qi et al. [49] proposed a combined process comprising liquid forging for billet preparation, isothermal forging, and solution treatment. Studies indicate that under optimal parameters (liquid forging with 1 wt.% Y, isothermal forging at 380 °C, and solution treatment at 520 °C), this process achieves excellent comprehensive properties—UTS of 315 MPa and an elongation to failure of 25.5—by regulating the transformation and spheroidization of I/W phase. Jiang et al. [92] developed Dual-Controlled Forming (DCF) technology, integrating a hydraulic forging system into a cold-chamber die-casting machine to synchronize complex shape forming with densification control. Immediately after high-speed injection filling, the forging unit applies 4000 kN of mechanical pressure to the partially solidified component. Research confirmed that DCF significantly refined primary α-Mg grains (average size < 50 μm) and completely eliminated micro-shrinkage cavities generated during die casting through mechanical feeding. Compared with conventional die casting alone, this hybrid technology boosts the UTS of AZ91D wheels by 38.3% (reaching 246 MPa) and increases elongation by 82.7% (reaching 9.5%). Additionally, Silva et al. [93] introduced KOBO extrusion technology for high-performance wrought alloys such as AZ80, combining linear extrusion with die rotational oscillation. This process induces superplastic deformation modes through periodic die twisting, reducing forming temperatures from the conventional 250–450 °C to below 200 °C (even room temperature) while achieving higher extrusion ratios (true strain $\epsilon_{1}$ ≈ 7). In the development of aerospace wheel hub shafts, this technology enabled precision forming of complex variable-wall-thickness components, providing crucial insights for manufacturing high-performance automotive wheels. Research confirms that AZ80 achieves a UTS of up to 350 MPa after extrusion. Concurrently, this process markedly enhances ductility, increasing the elongation of AZ61 and AZ31 to 18.4% and 35%, respectively. Testing demonstrates that components exhibit excellent stress distribution characteristics under 4000 N vertical and 1500 N lateral loads, ensuring service reliability under extreme operating conditions.

Building upon the foundation of macroscopic forming, the precise tailoring of microstructural features is paramount for optimizing the ultimate performance of the components. To mitigate prevalent defects encountered during the forming of large-scale AZ80 magnesium alloy components, Wang et al. [94] proposed the Expansion–Reduction Extrusion (ERE) process. Through multi-pass deformation and precise temperature control, this process achieved a uniform microstructure characterized by fine grains (approximately 28 μm) and atypical texture (*c*-axis deviation of about 60°), thereby improving the material’s inherent ductility. Subsequent post-deformation T5 aging treatment further revealed distinct property-evolution trajectories: extended aging significantly increased YS (from 185 MPa to 273 MPa) through precipitation strengthening, but concurrently caused substantial degradation in ductility (with the elongation to failure plummeting from 12% to 5%) and low-cycle fatigue life (decreased from 18,000 cycles to 8000 cycles). This study not only validates the efficacy of synergistic full-process design but, more crucially, elucidates the inherent strength–ductility and strength–fatigue trade-offs dictating the age-hardening response of the AZ80 alloy. The aforementioned studies demonstrate that, irrespective of the specific processing route, the fundamental essence lies in achieving the synergistic integration of shape forming and property optimization via thermo-mechanical processing coupled with dynamic microstructural evolution. In summary, combined forming processes, through multi-stage integration and coordination, can simultaneously address the dual challenges of mitigating macroscopic forming defects and tailoring microstructural features, thereby offering a highly promising paradigm for manufacturing high-performance magnesium alloy wheels.

## 5. Current Applications and Industrialization Challenges of Magnesium Alloy Automotive Wheels

### 5.1. Application Areas

Driven by increasingly stringent global automotive emissions regulations and the imperative of “dual carbon” targets, lightweighting has become a critical pathway for the automotive industry to achieve energy conservation, emissions reduction, and enhanced efficiency. Owing to their exceptional specific strength and stiffness, magnesium alloys are recognized as a pivotal material strategy for reducing vehicle mass and enhancing dynamic response. Currently, the application of magnesium alloy wheels is undergoing a strategic expansion from high-performance segments into the mass consumer market. Concurrently, forming technologies are diversifying, shifting from traditional forging toward advanced processes that offer higher efficiency and cost-effectiveness.

**Figure 21:**
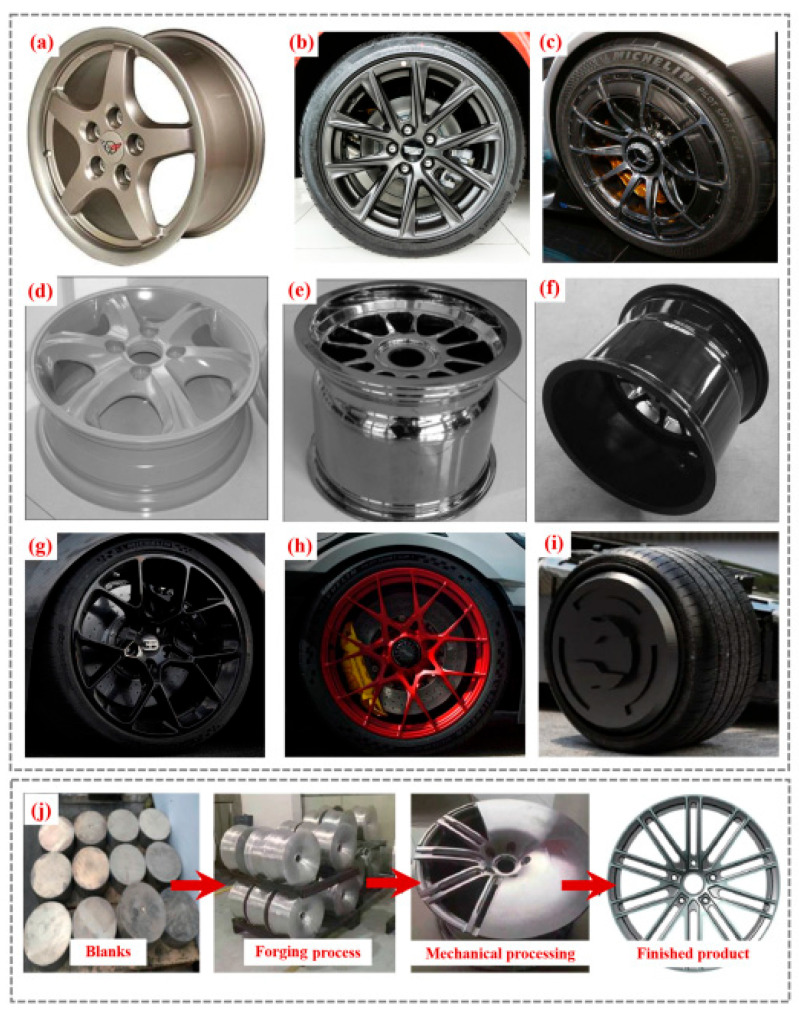
Development and application of magnesium alloy wheels [6]: (**a**) Chevrolet Corvette magnesium alloy wheel; (**b**) Cadillac CT4-V forged spin-formed magnesium alloy wheel; (**c**) AMG Project One biomimetic 9-spoke forged magnesium alloy wheel; (**d**) hollow billet extruded magnesium alloy wheel: (14 × 6) J; (**e**) hollow billet extruded magnesium alloy wheel: (13 × 6) J; (**f**) hollow billet extruded magnesium alloy wheel: (13 × 8) J; (**g**) Bugatti Chiron Super Sport 300+ Mg wheel; (**h**) Porsche 911 GT3 RS Mg forged wheel; (**i**) Bandit 9 electric race car Mg wheel; (**j**) forward–backward extrusion forming technology for magnesium alloy automotive wheels (Courtesy of Dingxin Magnesium Technology Co., Ltd. Zhuzhou, China).

Within the domain of high-performance automobiles, forged magnesium alloy wheels have emerged as a critical benchmark for evaluating vehicle dynamics. Figure 21 illustrates the diverse deployment of magnesium alloy wheels adopted across numerous models [6]. For instance, the forged magnesium alloy wheels fitted to the Porsche 911 GT3 RS (Figure 21h) reduce weight by approximately 8 kg compared to standard wheels [95]. This effectively lowers rotational mass and unsprung mass, thereby optimizing the suspension system’s dynamic response and traction performance. Similarly, the biomimetic forged wheels on the Bugatti Chiron Super Sport 300+ [96] (Figure 21g) and Mercedes-AMG Project One [97] (Figure 21c) underscore their pivotal role in performance-driven vehicles. Furthermore, the forged and spin-formed wheels on the Cadillac CT4-V (Figure 21b) and the magnesium alloy wheels on the Chevrolet Corvette [65] (Figure 21a) demonstrate the successful translation of this manufacturing technology into premium mass-production sectors. However, traditional forging processes remain constrained by inherent limitations such as complex multi-step processing routes, low material utilization, and high costs, which have hindered the widespread adoption of magnesium alloy wheels. To address these bottlenecks, innovations in forming technologies are providing critical support for their industrialization. Particularly, near-net-shape forming processes—represented by hollow billet extrusion (Figure 21d–f) and forward–backward extrusion (Figure 21j)—are being actively explored to enhance material utilization. Furthermore, more groundbreaking semi-solid injection molding technology has achieved significant progress in recent years. For instance, in 2025, Chongqing University and other institutions successfully prototyped the world’s first 16-inch semi-solid injection-molded magnesium alloy wheel, featuring a net mass of merely 7 kg—constituting an approximate 30% mass reduction relative to equivalent aluminum alloy products [98]. Furthermore, the 20-inch class wheel jointly developed by Dongfeng Motor and Shanghai Jiao Tong University not only achieved a 4.2 kg weight reduction per unit (a 35% reduction rate) but also increased material utilization to 85% while lowering comprehensive production costs by over 20%. This breakthrough overcomes the limitations of traditional processes for large-sized components [99]. Research indicates that this lightweighting solution is projected to extend the driving range of NEVs by 2–3%.

In summary, the current application landscape and technological evolution of magnesium alloy wheels underscore a pivotal transition. Driven by sustained innovation in alloy design and manufacturing methodologies, these components are consolidating their stronghold within high-performance sectors. Concurrently, by systematically dismantling traditional cost and scalability bottlenecks, they are paving the way for ubiquitous integration into the broader mass consumer automotive industry.

### 5.2. Industrialization Challenges

Currently, the large-scale industrialization of magnesium alloy wheels remains impeded by a series of critical challenges, including core bottlenecks such as material properties, manufacturing costs, process maturity, and long-term durability.

(1) Dual constraints of material performance and cost

In automotive wheel applications, magnesium alloys exhibit inherent limitations in absolute strength, fatigue resistance, and creep resistance. Reconciling the pursuit of extreme lightweighting with the strict prerequisites for structural integrity and stringent safety standards remains a formidable challenge. This demand for high performance compels the industry to continuously invest in R&D: on the one hand, developing new rare-earth or heat-resistant magnesium alloy formulations; on the other hand, establishing more rigorous non-destructive testing systems. Consequently, these requirements significantly escalate both the technical barriers and production costs. Furthermore, economics remain the core factor constraining market penetration. The raw material costs of magnesium alloys (particularly RE-based alloys), complex forming processes (such as precision forging and semi-solid injection molding), and surface protection treatments all significantly exceed those of traditional aluminum alloys. Although the energy-saving benefits from weight reduction are clear, the high cost of the final product remains the primary barrier to consumer acceptance and large-scale application. Therefore, minimizing the total lifecycle cost while ensuring service reliability represents a paramount challenge that the industry urgently needs to address.

(2) Manufacturing Processes and Scalability Bottlenecks

Traditional forging processes are hampered by complex multi-step processing routes and suboptimal material utilization, thereby limiting manufacturing throughput and cost control. Although breakthroughs in advanced technologies such as semi-solid injection molding (e.g., material utilization rates exceeding 85%) demonstrate potential for the near-net-shape forming of large, complex components and cost reduction, the maturity, production stability, and full industrial chain support for these processes still require further validation and refinement. This is essential to bridge the gap from “successful prototyping” to “stable, low-cost, large-scale mass production.”

(3) Technical Barriers to Durability and Corrosion Resistance

Magnesium alloys exhibit high chemical reactivity, making their corrosion resistance and long-term durability—particularly impact resistance and fatigue performance under complex road conditions and harsh environments—critical concerns for both industrial adoption and end-user confidence. This necessitates the development of more effective surface treatment technologies, protective coatings, or corrosion-resistant alloys, while simultaneously enhancing structural reliability. Mitigating these degradation mechanisms is an indispensable prerequisite for gaining broader market acceptance and securing a foothold in mainstream automotive platforms.

## 6. Conclusions and Outlook

Synthesizing the existing literature reveals that, driven by the imperatives of automotive lightweighting and low-carbon development, magnesium alloy wheels have emerged as a compelling lightweight structural solution. This is primarily attributed to their significant weight reduction potential (approximately 30% lighter than aluminum alloys), high damping capacity, and excellent thermal conductivity. Current research and industrial practice indicate the following key insights: In the context of alloy systems, cast magnesium alloys (such as AZ91D and AM60B) have been validated in small-scale applications due to their economic viability and superior castability. Meanwhile, wrought magnesium alloys (such as AZ80 and ZK61-Y), enhanced through plastic working and RE modification, exhibit superior mechanical properties and are poised to meet the demands of higher-performance wheels.

In terms of forming processes, traditional casting, plastic forming, and various combined forming techniques coexist. Research efforts are concentrated on utilizing advanced technologies—such as semi-solid processing and staged spinning—to refine microstructures and minimize defects, thereby enhancing the comprehensive performance of wheels. Notably, advanced techniques such as semi-solid injection molding have successfully increased material utilization to over 85%.

With respect to current industrial deployment, magnesium alloy wheels have achieved commercial breakthroughs in select high-performance vehicle models. However, the large-scale industrialization remains impeded by several persistent bottlenecks. Chief among these are prohibitive material costs, inadequate processing stability and microstructural reproducibility, and an inherent susceptibility to electrochemical degradation. Moving forward, the developmental trajectory of magnesium alloy wheels must converge on three strategic imperatives: performance augmentation, cost optimization, and high-volume manufacturability. To navigate these challenges, materials design must prioritize the development of application-specific alloy architectures and the exploration of low-cost compositional modifications to optimally reconcile the performance–cost dichotomy. Concurrently, manufacturing advancements should focus on the large-scale integration of advanced technologies augmented by computationally driven simulation and intelligent process controls, thereby guaranteeing microstructural homogeneity and production throughput. Finally, optimizing robust surface protection technologies and synergistic topological designs, coupled with enhanced industrial ecosystem collaboration, will prove indispensable for broadening their deployment across NEVs, commercial transport fleets, and other strategic sectors.

## Figures and Tables

**Table 1:** Weight comparison between magnesium alloy and aluminum alloy wheels [30].

Vehicle Type	Wheel Model/ Specification	Magnesium Alloy Wheel (kg)	Aluminum Alloy Wheel (kg)	Mass Reduction per Wheel (kg)	Total Mass Reduction (kg)
Sedans and Light Passenger Vehicles	5-1/2 JJ × 4	3.5–4.0	5.0–6.0	1.5–2.0	6.0–8.0
Mid-size Vehicles	6.0 GS × 16	8.0	11.5	3.5	21.0
Ten-wheel Heavy Trucks	7.5 V × 20	16.5	24.5	8.0	80.0
Ten-wheel Buses/Coaches	8.25 × 22.5	16.0	24.5	8.5	85.0

**Table 2:** Comparative Summary of Material Properties and Key Parameters for Magnesium Alloy Wheel Hubs.

Alloy	Process	Key Parameters	Key Performance	Ref.
AM50	LPDC	$T_{m}$: 390–410 °C; $T_{P}$: 705–710 °C	YS: 57.5 MPa; UTS: 193.8 MPa; EL: 9.1%	[54]
AZ91D	LPDC	$T_{P}$: 690 °C; $T_{m}$: 420 °C; $V_{p}$: 0.3 m/s;	Stress ↓ 14.4%; Strain ↓ 11%;	[33]
AM60B	VDC	$T_{P}$: 680 °C; $T_{pre}$: 250 °C; $V_{slow}$: 0.2 m/s; $V_{fast}$: 3.0 m/s	Stable filling; Eliminated macroscopic casting defects	[37]
AE81M + 1%Ca	LPDC	Melt holding *T*: 700 ± 20 °C	Weibull modulus rises markedly from 3.1 to 4.7	[39]
Mg-2.9Nd-0.18Zn-0.35Zr	SC	$T_{P}$: 665 °C; $T_{m}$: 225 °C; *P*: 80 MPa; T6 heat treatment	UTS: 305 MPa; YS: 165 MPa	[40]
Mg-2.96Nd-0.21Zn-0.39Zr	LPDC	$T_{P}$: 740 °C; $T_{m}$: 200 °C; T6 heat treatment	UTS: 287 MPa; YS: 152 MPa	[41]
AZ80	EF	Local deformation control	UTS: 339 MPa	[44]
AZ80 + 0.4%Ce	FE	ST: 415 °C/1.5 h; Two-stage aging: 120 °C/9 h + 175 °C/24 h	UTS: 364 MPa; YS: 295.36 MPa; EL: 10%	[47]
AZ80-LaMM	Single-pass radial forging	$T_{f}$: 400 °C; Upper die speed: 3 mm/s	DRX ↑; Uniform hardness; Weakened texture	[48]
ZK61-Y	LF	Liquid forging: 700 °C; $T_{m}$: 220 °C; Isothermal forging: 380 °C	UTS: 315 MPa; YS: 150 MPa; EL: 25.5%	[49]
AZ31	SF	Spinning temperature: 400 °C; Thinning rate: 70%	TS: 296.5 MPa; EL: 20.1%	[50]
AZ31-Ti (0.2–0.4%)	SC	$T_{P}$: 670–690 °C; *P*: 60–100 MPa	TS > 340 MPa	[53]

Note: $T_{p}$: Pouring temperature; $T_{m}$: Mold temperature; $T_{pre}$: Preheating temperature; $T_{f}$: Forging temperature; *V*: Velocity; *P*: Pressure; LPDC: Low-pressure die casting; VDC: Vacuum die casting; SC: Squeeze casting; EF: Extrusion forging; SF: Spin forming; FE: Forward extrusion; ST: Solution treatment; LF: Liquid forging; UTS: Ultimate tensile strength; YS: Yield strength; EL: Elongation; ↓: decrease; ↑: increase.

## Data Availability

No new data were created or analyzed in this study. Data sharing is not applicable to this article.
